# Evaluation of TaqMan qPCR System Integrating Two Identically Labelled Hydrolysis Probes in Single Assay

**DOI:** 10.1038/srep41392

**Published:** 2017-01-25

**Authors:** Alexander Nagy, Eliška Vitásková, Lenka Černíková, Vlastimil Křivda, Helena Jiřincová, Kamil Sedlák, Jitka Horníčková, Martina Havlíčková

**Affiliations:** 1 State Veterinary Institute Prague, Laboratory of Molecular Methods, Prague, 16503, Czech Republic; 2 National Institute of Public Health, National Reference Laboratory for Influenza, Prague, Czech Republic; 3 State Veterinary Institute Prague, Department of Virology and Serology, Prague, 16503, Czech Republic

## Abstract

Ongoing evolution of viral pathogens is a significant issue in diagnostic virology employing TaqMan qPCR/RT-qPCR. Specific concerns are related to false negativity due to probe binding failure. One option for compensating for such deficiency is to integrate a second identically labelled probe in the assay. However, how this alteration influences the reaction parameters has not been comprehensively demonstrated. In the present study, we evaluate a TaqMan protocol using two identically labelled hydrolysis probes (simple, LNA (locked-nucleic-acid)) and MGB (minor-groove-binder) modified probes and combinations thereof in a single assay. Our results based on a synthetic amplicon suggest that the second probe does not compromise the TaqMan qPCR/RT-qPCR parameters, which repeatedly and reproducibly remained comparable to those of the corresponding single-probe assays, irrespective of the relative probe orientation, whether opposite or tandem, and probe modifications or combinations thereof. On the other hand, the second probe additively contributed to the overall fluorescence signal. The utility of the dual-probe approach was demonstrated on practical examples by using field specimens. We hope that the present study might serve as a theoretical basis for the development or improvement of TaqMan qPCR/RT-qPCR assays for the detection of highly variable nucleic acid templates.

Real-time PCR utilising 5′ nuclease or hydrolysis probes^1^, also known as TaqMan qPCR, is an essential and powerful tool used in various areas of life science. It also has great potential in diagnostic microbiology, where the TaqMan qPCR is often the first-line screening method for the detection of many viral or bacterial pathogens in human, animal or plant specimens.

The TaqMan qPCR employs a pair of primers and a non-extendable probe. The probe is a short, sequence specific oligonucleotide, which binds within the region delimited by the primers. One end, usually the 5′ terminus, of the probe is labelled using a fluorescent dye, while the other one, the 3′ end, is tagged with a quencher. The fluorescent dye and the quencher form a donor-acceptor FRET (fluorescence resonance energy transfer) pair^2^. As the amplification proceeds, the TaqMan probe hybridizes to the target sequence downstream of one of the primers. These two oligonucleotides form an effective unit. Once the upstream primer begins to be extended, the *Taq* polymerase mediated 5′→3′ hydrolysis of the probe takes place, leading to the disruption of the FRET pair accompanied by dye release from the quenching effect. Subsequent illumination of the reaction mix results in detectable fluorescence with a dye-specific wave-length. The emitted fluorescence signal is proportional to the amount of accumulated PCR product.

Conventionally, the TaqMan qPCR is a tri-oligonucleotide system. The probe, as the third oligonucleotide, introduces an additional level of sequence specificity into the assay. On the other hand, it also substantially contributes to the susceptibility of the technique to template mutations. Mismatches in the probe-binding region decrease or completely eliminate the probe binding, leading to false negativity. This is an important and frequently reported issue in diagnostic virology^3^,^4^,^5^,^6^,^7^,^8^,^9^,^10^,^11^,^12^,^13^,^14^ that is faced in the detection of highly variable viral nucleic acid (NA) templates. Therefore, it is crucial to design novel approaches to improve the inclusivity and to reduce the probability of false negative results of diagnostic TaqMan qPCR assays.

One option for improvement comes from the TaqMan principle itself. The parsing of the reaction mechanism in more detail suggests that, in addition to effective pair-mediated signal generation, the second primer-induced opposite strand synthesis runs in parallel. Since this process is not captured by fluorescence imaging, the opposite strand remains invisible. Hence, the signal observed during the TaqMan qPCR is generated in fact upon only 50% of the accumulated amplicon strands. Visualization of the “invisible” amplicon fraction by coupling the second primer with a hydrolysis probe, or more generally by including more distinct probes amidst the forward and reverse primers, could therefore significantly reduce the probability of probe binding failure and improve the inclusivity of a given assay.

Nevertheless, TaqMan RT-qPCR assays utilizing two or more identically or differently labelled probes^15^,^16^,^17^ are rare in diagnostic microbiology. The probable reason why this approach was not established is the absence of a more comprehensive evaluation background. Specifically, it is not clear how the identically labelled probes operating alongside one another in a single assay influence the basic reaction parameters, like the Cq, sensitivity, efficiency and overall fluorescence. Does the second probe alter them? Can we combine identically labelled probes with different modifications like LNA and MGB in the same assay? Is there a difference between the probes in opposite and tandem orientations?

To answer these questions, and to propagate the application of the dual-probe TaqMan approach, in the present study, we evaluate a TaqMan qPCR protocol using two identically labelled hydrolysis probes binding to discrete, non-overlapping template regions in a single qPCR assay. Based on a synthetic DNA template construct, we investigate the effect of the dual-probe assay on the basic reaction parameters, including the Cq, relative fluorescence intensity, efficiency, repeatability, reproducibility and sensitivity. All of the parameters were evaluated for simple (unmodified) probes, LNA or MGB modified probes, and combinations thereof either in opposite or tandem orientations. Finally, the utility of the presented approach in diagnostic qPCR was demonstrated on practical examples.

## Results

### Nucleic Acid Standard Design

Theoretically, there are two options for how to integrate a second hydrolysis probe into the TaqMan assay: (i) in opposite orientation, i.e. downstream of the second primer, thus creating a second effective unit (primer-probe pair), the two units then each bind on one template strand; or (ii) in tandem or serial orientation, where the probes bind consecutively downstream of one of the primers.

**Figure 1:**
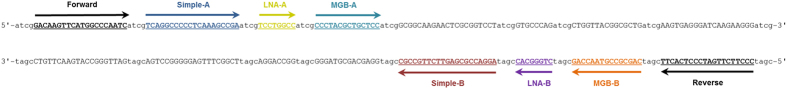
Schematic representation of synthetic nucleic acid standard. The 162 nucleotides-long sequence starts with a four-nucleotide tag, followed by a pair of primers (in black, underlined). The amplicon contains six probe binding motifs, three located downstream of the forward primer and three located downstream of the reverse primer. For abbreviations and further details, please refer to Fig. 2.

**Figure 2:**
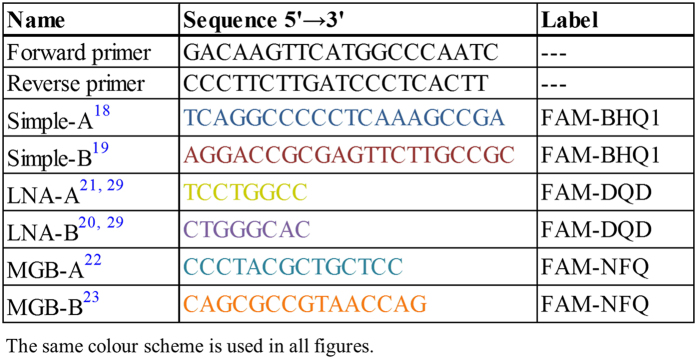
The primer and probe sequences used in the study. LNA locked nucleic acid, MGB minor groove binder, FAM 6-Carboxyfluorescein, BHQ black hole quencher, NFQ non fluorescent quencher, DQD dark quencher dye.

To test both options, a synthetic NA standard was designed (Fig. 1). The standard sequence starts with a short tag, followed by a pair of primers delimiting the amplicon. The amplicon encompasses six probe-binding motifs, three located downstream of the forward primer and three located downstream of the reverse primer. The probe sequences (Fig. 2) were selected from the previously published assays so as to enable the testing of simple and LNA and MGB modified probes in various combinations, all labelled with a FAM reporter dye. So, each consecutive triplet contains one simple (Simple-A^18^, Simple-B^19^), one LNA modified (LNA-A^20^, LNA-B^21^), and one MGB modified (MGB-A^22^, MGB-B^23^) probe motif.

The designed NA standard sequence enabled nine different assays with the probes in opposite orientations to be set up: 1. simple-simple, 2. LNA-LNA, 3. MGB-MGB, and simple-LNA, simple-MGB, and LNA-MGB pairs, each in two permutations. In addition, it was possible to set up six probes in tandem orientations (simple-LNA, simple-MGB, and LNA-MGB, each in two permutations). This provided numerous testing opportunities, with only the combinations most likely to be applied being further evaluated.

### Evaluation of Reaction Parameters of Dual-probe TaqMan qPCR Assay

**Figure 3:**
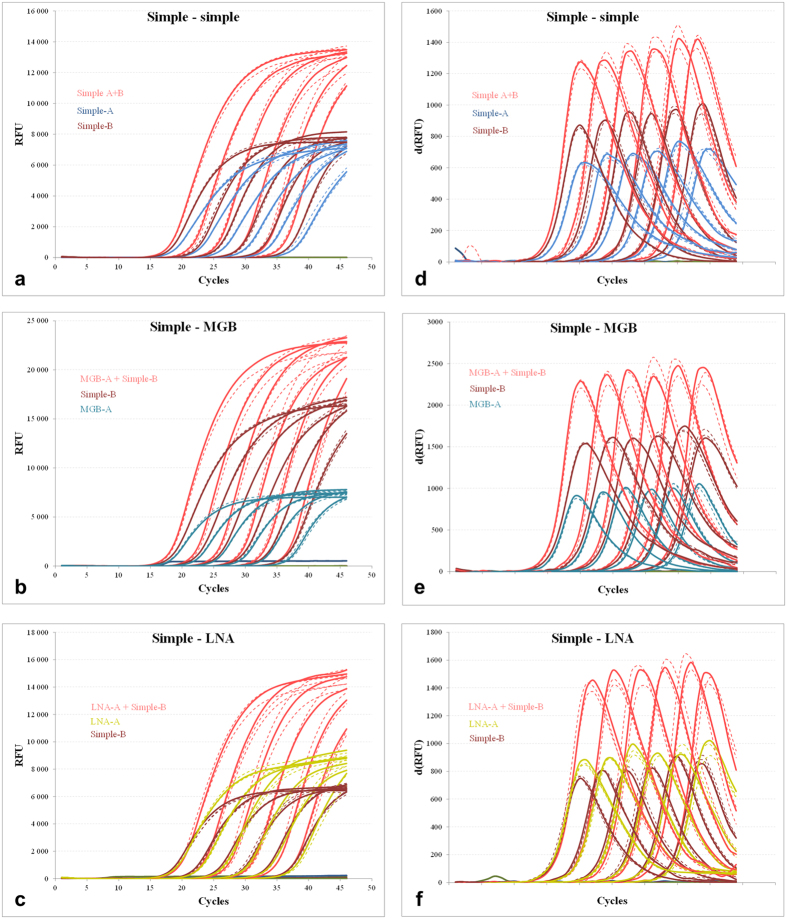
Dilution curve analysis of dual-probe TaqMan qPCR assays. Dilution curves **(a–c)**, arranged from 1e2 to 1e7 NA standard copies per μl of template in three replicates, of the simple-simple, simple-MGB, and simple-LNA dual-probe assays in opposite orientations and their first derivative **(d–f)**. The dual-probe assays are highlighted in red and the corresponding single-probe counterparts in probe-specific colours. For clarity, certain curves were dashed. The probe sequences and their schematic representation and colouring are shown in Figs 1 and 2. For the data regarding additional dual-probe assays, either in opposite or tandem orientations, please refer to Supplementary Information 1, Figure S1.1 and Supplementary Information 2, Figure S2.1, respectively.

**Figure 4:**
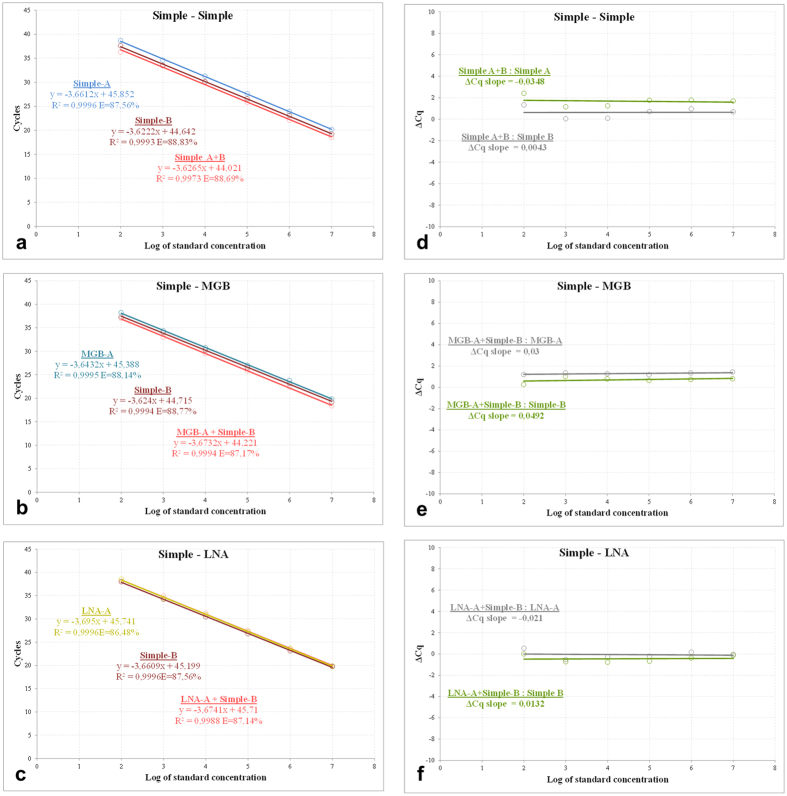
Calibration and ΔCq curve analysis of dual-probe TaqMan qPCR assays. Calibration **(a–c)** curves of the simple-simple, simple-MGB and simple-LNA dual-probe assays in opposite orientations. The curves were prepared for a dilution gradient from 1e2 to 1e7 NA standard copies per μl of template in triplicates. The dual-probe assays are highlighted in red and the corresponding single-probe counterparts in probe-specific colours. The probe sequences and their schematic representation and colouring are shown in Figs 1 and 2. The ΔCq curves **(d–f)**, representing the Cq differences between the dual-probe versus the corresponding A and B assays at each concentration point, were coloured in green and grey, respectively. For the data regarding additional dual-probe assays, either in opposite or tandem orientations, please refer to Supplementary Information 1, Figure S1.2 and Supplementary Information 2, Figure S2.2, respectively.

Six combinations of probes in opposite orientation: 1. simple-simple, 2. simple-MGB, 3. simple-LNA (Fig. 3 Panels a–c) and 4. MGB-MGB, 5. LNA-LNA, and 6. LNA-MGB (Supplementary Information 1, Fig. S1.1, Panels a–c), were selected to test the opposite dual-probe qPCR assay. The tandem orientations were investigated in three probe combinations: 1. simple-MGB, 2. simple-LNA, and 3. LNA-MGB (Supplementary Information 2, Fig. S2.1, Panels a–c). Dilution and calibration curves covering a quantitative range of six magnitudes (from 1e2 to 1e7 NA standard copies per μl of template), were prepared to evaluate the reaction parameters. The dilution curves showed a parallel course for the particular dual-probe and the corresponding single-probe subsets in the early exponential phase of amplification. This resulted in highly similar Cq values, supported by low standard deviation (SD) across the entire dilution gradient, irrespective of the combination of the hydrolysis probe modifications or the tested orientations thereof. The close Cq values were also reflected in the parallel and overlapping calibration curves, where the corresponding assays had comparable amplification efficiencies (Fig. 4, Panels a–c and Supplementary Information 1, Fig. S1.2, Panels a–c, and Supplementary Information 2, Fig. S2.2, Panels a–c). Similarly, the ΔCq straight lines showed parallel courses, with the slope values significantly below the 0.1 threshold (Fig. 4, Panels d–f and Supplementary Information 1, Fig. S1.2, Panels d–f and Supplementary Information 2, Fig. S2.2, Panels d–f), again suggesting that the dual-probe and the corresponding single-probe TaqMan assays have comparable efficiencies. One slight deviation in the data was observed. In particular, the opposite MGB-A+LNA-B assay (Supplementary Information 1, Fig. S1.2, Panel c) showed slightly lower efficiency than the corresponding MGB-A subset. This was also reflected in the elevated ΔCq slope of 0.157 (Supplementary Information 1, Fig. S1.2, Panel f), which, though small, is still higher than the 0.1 threshold. However, the observed deviation was attributed to experimental imperfections and did not contradict the general findings of the study.

Taken together, the calibration curve and ΔCq slope data clearly proved that the second identically labelled probe did not significantly influence the TaqMan qPCR assay. The dual-probe assays stably showed highly similar parameters, in comparison with the single-probe counterparts, independently of the probe modifications, orientations or combinations tested.

### Evaluation of Reaction Fluorescence of Dual-probe TaqMan qPCR Assay

As was expected, the second probe in the TaqMan qPCR assay had an additive effect on reaction fluorescence. To quantitatively express the observed differences, the steepness and the first derivative maxima of the amplification curves were compared between the corresponding subsets across the entire dilution gradient (Fig. 3, Panels d–f and Supplementary Information 1, Fig. S1.1, Panels d–f and Supplementary Information 2, Fig. S2.1, Panels d–f). The comparison revealed that the opposite probe assays and the tandem dual-probe assays had both steeper and brighter amplification curves (15 to 60% and 20 to 43%, respectively) than the corresponding single-probe counterparts (Supplementary Information 1, Table S1.1 and Supplementary Information 1, Table S2.1). The observed differences in fluorescence were retained across the entire dilution gradient, regardless of the probe combination tested.

### Repeatability and Reproducibility of Dual-probe TaqMan qPCR Assay

The intra-assay variation (i.e. repeatability) was tested on three different concentrations (high, 1e6; medium, 1e4; and low, 1e2 NA standard copies per μl of template, each in triplicate) in a single run.

Evaluation of the mean Cq and SD of the particular standard triplicates showed SD values to be lower than or equal to 0.41 for all six opposite dual-probe TaqMan assays (Supplementary Information 1, Table S1.2). The three tandem-probe assays exhibited SD ≤ 0.56. These results indicated low spread from the mean and thus low intra-assay variation within all investigated runs (Supplementary Information 2, Table S2.2).

The inter-assay variation (i.e. reproducibility) was tested by three operators on three CFX 96 instruments using that same standard set. The mean Cq, SD, and %RSD values were as follows: SD ≤ 0.67 and %RSD ≤ 2.36% for the opposite dual-probe TaqMan assays and SD ≤ 0.52 and %RSD ≤ 1.60% for the tandem-probe assays (Supplementary Information 1, Table S1.3 and Supplementary Information 2, Table S2.3). The results proved the high degree of precision of the data and low inter-assay variation.

### Sensitivity of Dual-probe TaqMan qPCR Assay

**Figure 5:**
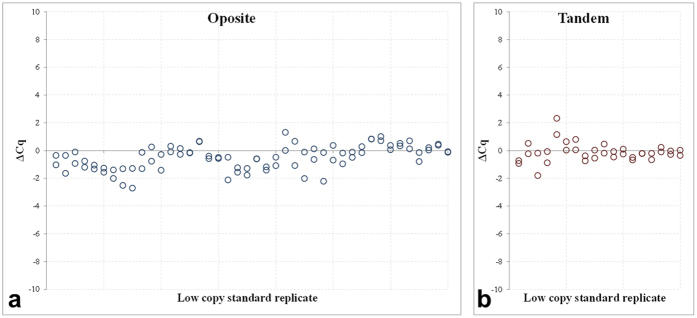
Sensitivity of dual-probe TaqMan qPCR assays. The sensitivity of the opposite (**a**, n = 2 × 42) and tandem (**b**, n = 2 × 18) dual-probe TaqMan qPCR was tested by comparing the ΔCq values relative to the corresponding single-probe assays in six low copy NA standard dilutions, i.e., to obtain Cq values ≥ 30. (a) Generalises the results of six probe combinations: simple-simple, simple-MGB, simple-LNA (two data sets), MGB-MGB, LNA-LNA, and LNA-MGB, and (**b**) shows the ΔCq of three probe combinations: simple-MGB, simple-LNA, and MGB-LNA, respectively.

The results of the sensitivity analyses are summarised in Fig. 5a and b. Overall, the ΔCq scatter plot, including 2 × 42 paired analyses for all opposite probe combinations, showed predominant distribution of the ΔCq values below the diagonal, or more specifically, within the interval of 0 and −2 (dual-probe mean ΔCq 35.58 ± 1.89 versus 36.19 ± 1.91 and 35.97 ± 2.01). The tandem dual-probe assays, represented by 2 × 18 paired analyses exhibited symmetrical distribution of the particular ΔCq values across the diagonal (dual-probe mean ΔCq 35.28 ± 1.80 versus 35.52 ± 2.05 and 35.28 ± 1.93).

The results sufficiently proved that including the second identically labelled probe into the TaqMan qPCR does not compromise the assay sensitivity, with Cq values remaining virtually identical, even at limited template concentrations. This was observed irrespective of the probe modification, combination or orientation.

Finally, the influence of the second probe on the FAM signal and data interpretation at limited standard concentrations was investigated. The %d(RFU) scatter plots (see methods) consistently expressed stronger FAM signal across the entire panel, with the data distribution between 10–60% for all tested probe combinations.

### Demonstration of Utility of Dual-probe TaqMan qPCR in Diagnostic Microbiology

**Figure 6:**
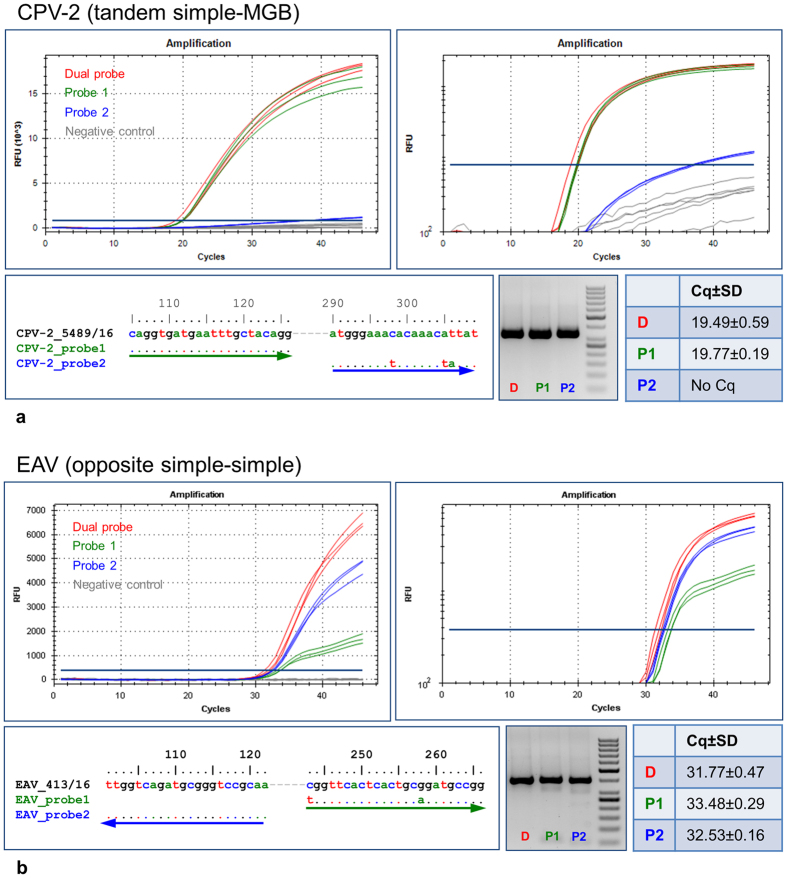
Demonstration of utility of dual-probe TaqMan qPCR in diagnostic microbiology. The amplification curves of the dual-probe CPV-2 qPCR (tandem simple-MGB configuration, (**a**) and EAV RT-qPCR (opposite simple-simple configuration, (**b**), and the related single-probe assays (Supplementary Information 3, Table S3.1), shown in classical and logarithmic representation. All reactions were performed in triplicates. The dual-probe assays were highlighted in red and the probe1 and probe2 assays with green and blue, respectively. Each analysis was supplemented with the probe-to-template sequence alignment, with arrows in corresponding colours indicating the probe directions. The electrophoreograms compare the amplification products (one replicate), and the tables the mean Cq values between the assays. Additional examples were shown in Supplementary Information 3, Figure S3a–c.

The utility of the dual-probe approach in improving the diagnostic TaqMan qPCR was demonstrated on two assays: Equine arteritis virus (EAV) RT-qPCR and Canine parvovirus 2 (CPV-2) qPCR (Supplementary Information 3, Table S3.1). The EAV assay used simple-simple probes in opposite orientation, while the CPV-2 assay utilized a simple-MGB tandem-probe pair. To investigate the mutual compensatory effect of the probe pairs during the amplification, various EAV and CPV-2 strains holding different nucleotide mutations that weakened or knocked out one of the probes were selected. The most striking examples were shown in Fig. 6.

A dual-probe (tandem simple-MGB configuration) CPV-2 qPCR and the corresponding single-probe assays were used in triplicates to investigate a CPV-2 strain, which perfectly matched the first probe but held three nucleotide changes in the second probe binding site. One of these changes was located approximately in the middle, while the last two were situated close to the MGB-modified 3′ end of the probe. The results revealed that the observed mutations were deleterious to the CPV-2_probe2 binding and hydrolysis. Hence, the CPV-2_probe2 qPCR assay failed to detect this CPV-2 strain despite the presence of the underlying amplicon, as was suggested by electrophoresis. On the contrary, the dual-probe assay showed clear CPV-2 positivity with virtually identical parameters to the CPV-2_probe1 counterpart. This indicated that in the dual-probe setting, the CPV-2_probe1 sufficiently compensated for the CPV-2_probe2 deficiency.

In the second example, a dual-probe EAV RT-qPCR assay (opposite simple-simple configuration) was used, along with the single-probe counterparts, to detect an EAV strain with 100% similarity to the EAV_probe2, while carrying two mutations relative to the EAV_probe1. The first of these mutations was located at the extreme 5′ end, suggesting possible interference with hydrolysis. The second change was slightly eccentric and shifted towards the 3′ end. Interestingly, as observed on the amplification plot, the EAV_probe2 probe was still capable of detecting the tested EAV strain, but with weak FAM signal gain resulting in a flat amplification curve. Nevertheless, even this weak fluorescence yield can contribute to the overall signal of the dual-probe assay, which outperformed the perfectly matching single-probe assay.

Additional examples of the dual-probe CPV-2 and EAV assays were provided in Supplementary Information 3, Figure S3a–c.

## Discussion

Ongoing evolution of viral pathogens is a significant issue in diagnostic virology employing TaqMan qPCR/RT-qPCR. Specific concerns are related to false negativity as a result of decreased probe binding efficiency or complete binding failure due to mutations in the corresponding template regions^3^,^4^,^5^,^6^,^7^,^8^,^9^,^10^,^11^,^12^,^13^,^14^. Hence, even if the specific amplicons are generated, the assays remain non-fluorescent, thus suggesting no amplification in a TaqMan qPCR setting. Including a second probe in the assay may therefore compensate for the deficiency of the first one and restore the assay inclusivity. There are several options for how to integrate the second probe into the TaqMan qPCR assay. The probes can bind to the complementary amplicon strand or consecutively downstream of one of the primers. Further, they can be simple or additionally modified to increase the melting temperature. Finally, the probes can hold identical or different fluorescent labels.

Our results, based on a synthetic NA construct, suggested that the integration of two identically labelled hydrolysis probes in an equimolar ratio does not compromise the basic parameters of the TaqMan qPCR/RT-qPCR. The dual-probe assays repeatedly and reproducibly retained comparable Cq, reaction efficiency and sensitivity to the corresponding single-probe counterparts. In addition, these parameters were not influenced by the relative probe orientations, whether opposite or tandem, nor were they affected by probe modifications or combinations.

The capacity of the dual-probes to mutually complement one another in real conditions was demonstrated on two distinct assays, CPV-2 qPCR with tandem simple-MGB, and EAV RT-qPCR with opposite simple-simple configurations. The templates were CPV-2 and EAV positive field specimens with various mismatches in one of the probe binding regions, which weakened or completely eliminated the hybridization. Unfortunately, we had no viral strains in our repository holding partially deleterious mutations in both of the probe binding sites. Hence, the effect of such mutations remains to be elucidated.

As was shown, the perfectly matched probes were able to fully compensate for the decreased efficiency or the complete failure of the second one. In spite of this, the weakened probes were able to contribute to the overall fluorescence signal, which facilitates the assay evaluation. All of these conclusions agreed with our initial conclusions based on the synthetic DNA construct. However, we would like to emphasize that the CPV-2 and EAV assays were set up for demonstration purposes only, and their use for routine screening would require optimization and comprehensive validation, including a broad panel of negative specimens to exclude interference between the probes and generation of false positive signals.

Although the general results of our study agreed well with the previous findings of Yip and colleagues^15^, the Cq and efficiency values of the dual-probe assays are slightly contradictory. Specifically, we did not find as significant improvement in Cq and efficiency values of the dual-probe assays across our data set as reported by Yip and colleagues^15^. However, the theory of qPCR does not suggest significant improvements in these parameters either. In tandem orientations, the two probes bind to one of the DNA template strands. Assuming that the tandem-probe and the corresponding single-probe assays exhibit comparable efficiencies, the recognised template amount in each cycle is identical, i.e. 50%, since in both cases the probe binding and hydrolysis take place upon a single strand of a double-strand template. Hence, all the assays would have identical Cq values and the differences would only be reflected in the higher fluorescence yield of the tandem-probe assays. On the contrary, a dual-probe assay with opposite probe configuration simultaneously detects both strands of the template during each single cycle. Indeed, there is a 2-fold difference between the opposite and tandem-probe assays, which would theoretically yield lower Cq values. Nevertheless, the theory further suggests that if a qPCR is 100% efficient, the 2-fold measurements differ by one cycle between the means^24^. However, in reality, the amplification efficiency is never absolute, and therefore, the expected one cycle difference between the 2-fold dilutions is reduced. In addition, it is burdened with higher experimental variations^25^. Hence, the Cq improving-effect of the second probe in opposite orientation is difficult to distinguish. In summary, there is no obvious theoretical explanation for the observed contradictions. Instead, it seems that the Cq and reaction efficiency improvements observed by Yip and colleagues^15^ resulted from sequence mismatches in the first probe and the compensatory effect of the second one. This option was not further investigated.

On the other hand, the dual-probe approach greatly improved the reaction fluorescence. In agreement with Yip and colleagues^15^, the second identically labelled probe had an additive effect on the overall reaction fluorescence, which increased it by up to 60% in comparison with the single-probe assays. This represents a significant improvement which facilitates the evaluation and interpretation of weak positive specimens. Further, identically labelled FAM probes enable pre-existing internal controls to be used without the necessity of re-labelling. Finally, they provide the option of advanced analyses^14^, and their use is cost-effective.

However, the major advantage of the dual-probe approach does not lie in the improvement of the reaction parameters itself, but in the ability to increase the inclusivity of the diagnostic TaqMan assay. The second probe would extend or restore the potential of the pre-existing diagnostic assays to detect novel genetic variants. This potential is of utmost importance in universal assays, which are often used as the first-line screening tool in diagnostic microbiology^20^,^26^,^27^,^28^.

Universality should also be kept in mind during the development of any novel TaqMan screening assay. To meet this requirement, the primers and the probes have to be selected at the most conserved regions of the targeted genome. Optionally, evolutionarily conserved regions are identified by multiple sequence alignment (MSA), including sequences from a broad time period and from different geographic regions and host species. However, the more diverse the sequence collection of the MSA, the less abundant the conserved positions are. So, once the primers have been selected, the MSA may not provide additional evolutionarily conserved positions suitable for probe selection. The advancement of the dual-probe TaqMan strategy is to enable probe selection at semi-conserved motifs, where the two probes may complement or overlap one another in sequence inclusivity. This might facilitate the development of novel screening assays. It is worth bearing in mind, however, that the opposite dual-probe system is inherently predisposed towards lower Cq values and should be the preferred choice during assay design.

Despite the indisputable advantages, the dual-probe TaqMan technique has several drawbacks. Primarily, the second probe introduces an additional level of complexity into the assay. Therefore, the dual-probe assay requires more comprehensive optimization, evaluation and validation than a single-probe TaqMan assay. Attention should mainly be paid to the generation of nonspecific fluorescent signals, which should be tested on a panel of negative specimens, as well as non-template controls. Further, when one of the probes is knocked out, it cannot be distinguished at first glance which one was influenced. The probe failure is reflected only in the drop of the overall signal intensity of the assay. This requires more attention during the interpretation of the results. Additional disadvantages are the increased reaction cost and possibly an extra pipetting step during the master mix preparation. However, in spite of the negative aspects mentioned above, we are firmly convinced that the dual-probe strategy using identically labelled probes may serve as a valuable option in the development or improvement of a TaqMan qPCR/RT-qPCR assay for the detection of highly variable nucleic acid templates.

## Methods

### Field Specimens

Canine parvovirus 2 (CPV-2) positive canine organ suspensions and Equine arteritis virus (EAV) positive equine semen specimens were selected for analysis. None of the specimens were initially collected for the purpose of this study. The ethical standards in animal welfare and protection are subject to inspection by the State Veterinary Administration of the Czech Republic.

### Nucleic Acid Extraction

Total NA was extracted using the MagNAPure Compact (Total NA Extraction Kit I, Roche), with input sample volumes from 200 or 400 μl and elution volume of 50 μl.

### Nucleic Acid Standard

A synthetic DNA construct (Fig. 1, Integrated DNA Technologies) in a 4 nM scale was diluted in 1 ml of nuclease free water (Qiagen). The copy number/μl of the stock was then calculated and used for downstream experiments. The primers and unmodified dually labelled FAM-BHQ1 hydrolysis probes (Fig. 2) were supplied by Generi Biotech, Czech Republic. The MGB probes (Life Technologies) incorporated a FAM reporter dye and a 3′ nonfluorescent quencher (NFQ), with the MGB moiety attached to the quencher molecule. The LNA probes, UPL#104 and UPL#162, were FAM and dark quencher dye (DDQ) labelled octamers selected from the 165 Universal Probe Library (Roche)^29^.

### Dual-probe TaqMan qPCR

All of the reaction mixes were prepared using QuantiTect Probe PCR or QuantiTect Probe RT-PCR Kits (Qiagen), with a 0.6 μM of primers and 0.2 μM of each probe in a final volume of 12.5 μl (10.5 μl reaction mix and 2 μl of DNA template), using white opaque and foil-sealed plates (Bioplastics). The primers and the probe sequences for the DNA standard are listed in Fig. 2. The qPCR thermoprofiles for the synthetic DNA construct universally started with an initial activation at 95 °C for 15 min, followed by 45 cycles of 95 °C for 10 s and 60 °C for 30 s with a signal acquisition in the FAM channel at the end of the annealing/extension step.

The dual-probe CPV-2 qPCR assay consisted of a tandem simple-MGB probes, amplifying a 340 bp-long amplicon with the forward primer and first probe according to Streck end colleagues^30^, and the reverse primer and the second probe according to Decaro end colleagues^31^. The thermo-profile started at 95 °C for 15 min, followed by 45 cycles of 95 °C for 10 s and 60 °C for 1 min, with a signal acquisition in the FAM channel.

The dual-probe EAV RT-qPCR assay delimited a 399 bp-long region^32^ and included a pair of simple probes^32^,^33^ in opposite orientation. The thermo-profile started with reverse transcription of 50 °C for 30 min and 95 °C for 15 min, followed by 45 cycles of 95 °C for 20 s, 60 °C for 1 min and 72 °C for 30 s, with a signal acquisition in the FAM channel at the end of the annealing/extension step. The primers and the probe sequences for the CPV-2 and EAV assays are listed in Supplementary Information 3, Table 3.1.

All of the reactions were run in triplicates on a CFX 96 (BioRad) thermal cycler. The Cq values were estimated by analysing the data as a single pool, using the automatic threshold and baseline cycle option of the CFX Manager Software v3.1. The reactions of interest were subjected to electrophoretic analysis by using ethidium bromide-containing 2% agarose gel in a TAE buffer and 10 V/cm.

### Sequence Analysis

The amplicons of interest were purified by using a High Pure PCR Purification Kit (Roche). Sequencing reactions were prepared using a Big Dye Terminator Cycle Sequencing Kit v3.1 and evaluated on 3130 or 3500 Genetic Analysers (all from Life Technologies). The sequences were aligned manually. The graphical alignment was generated in a BioEdit sequence alignment editor^34^.

### Evaluation of Reaction Parameters of Dual-probe TaqMan qPCR Assay

Dilution and calibration curves were constructed on the basis of a 10-fold dilution gradient of the synthetic DNA standard, covering a quantitative range of six magnitudes (from 1e2 to 1e7 NA standard copies per μl of template) in triplicates including three non-template controls. To evaluate the effect of the second probe on the TaqMan reaction, each individual run was set up in three subsets. The first subset represented the dual-probe reaction (0.2 μM of each probe), while the second and third ones contained the corresponding single probes (0.2 μM) in separate reactions. For each subset, the efficiency E, slope *k,* and the equation and R^2^ values of the calibration curve regression line were determined.

To infer the comparability of the two probe assays with the single-probe counterparts, the ΔCq slope approach was used^35^. First, the ΔCq between the dual-probe and corresponding single-probe reactions was calculated according to the formula: ΔCq = Cq(dual-probe) - Cq(simple-probe), and plotted against the logarithm of the template concentration. Then, the slopes of the resulting straight lines were compared between the reactions. If the slope values were <0.1, the reaction efficiencies were comparable.

### Evaluation of Reaction Fluorescence of Dual-probe TaqMan qPCR Assay

The relative fluorescence unit (RFU) values of the amplification curves gathered in the FAM channel were compared between the subsets. We defined two parameters: the d(RFU) and the steepness *k*. The d(RFU) represents the fluorescence value at a specific point of the sigmoidal trajectory, which corresponds with its first derivative maximum. The steepness was defined as the slope of the linear regression line driven along the exponential region of the amplification curve. The exponential region was determined from those RFU data points that provided the highest correlation (R^2^ > 0.995) of the regression line.

### Repeatability and Reproducibility of Dual-probe TaqMan qPCR Assay

The repeatability or intra-assay variation of the two probe TaqMan assays was estimated from the mean Cq and standard deviation (SD) values obtained from three dilutions of the NA standard, 1e2, 1e4, and 1e6 copies per μl of template in three replicates in a single run.

The reproducibility, i.e. inter-assay variation of the two probe TaqMan assays was inferred from three independent runs of the above three NA standard dilutions prepared by three distinct operators and run on three different CFX 96 instruments. Then, the mean Cq, SD, and relative standard deviation as a percentage (%RSD, also known as the coefficient of variation) were calculated.

### Sensitivity of Dual-probe TaqMan qPCR Assay

The relative sensitivity of the dual-probe TaqMan qPCR assays was tested on six opposite probe and three tandem-probe assays by using various NA standard dilutions to reach Cq values ≥ 30. The particular ΔCq values between the corresponding dual-probe and single-probe assays were estimated according to the above formula and compared.

## Additional Information

**How to cite this article:** Nagy, A. *et al*. Evaluation of TaqMan qPCR System Integrating Two Identically Labelled Hydrolysis Probes in Single Assay. *Sci. Rep.* **7**, 41392; doi: 10.1038/srep41392 (2017).

**Publisher's note:** Springer Nature remains neutral with regard to jurisdictional claims in published maps and institutional affiliations.

## Supplementary Material

Supplementary Information
