# Supplementary material for: Evaluation of TaqMan qPCR System Integrating Two Identically Labelled Hydrolysis Probes in Single Assay
Source: Sci Rep. 2017 Jan 25;7:41392. doi: 10.1038/srep41392 (PMC5264587; doi:10.1038/srep41392)
Supplement: Supplementary Information [file srep41392-s1.pdf]

Evaluation of TaqMan qPCR System Integrating Two Identically Labelled Hydrolysis Probes in Single Assay

Alexander Nagy[1,3*], Eliška Vitásková[1], Lenka Černíková[1], Vlastimil Křivda[1,2], Helena Jiřincová[3], Kamil Sedlák[2], Jitka Horníčková[2], Martina Havlíčková[3]

[1] State Veterinary Institute Prague, Laboratory of Molecular Methods, Prague, 16503, Czech Republic

[2] State Veterinary Institute Prague, Department of Virology and Serology, Prague, 16503, Czech Republic

[3] National Institute of Public Health, National Reference Laboratory for Influenza, Prague, Czech Republic

Supplementary Information 1. Results of Dual-probe TaqMan qPCR Assays with Opposite Probe Orientations.

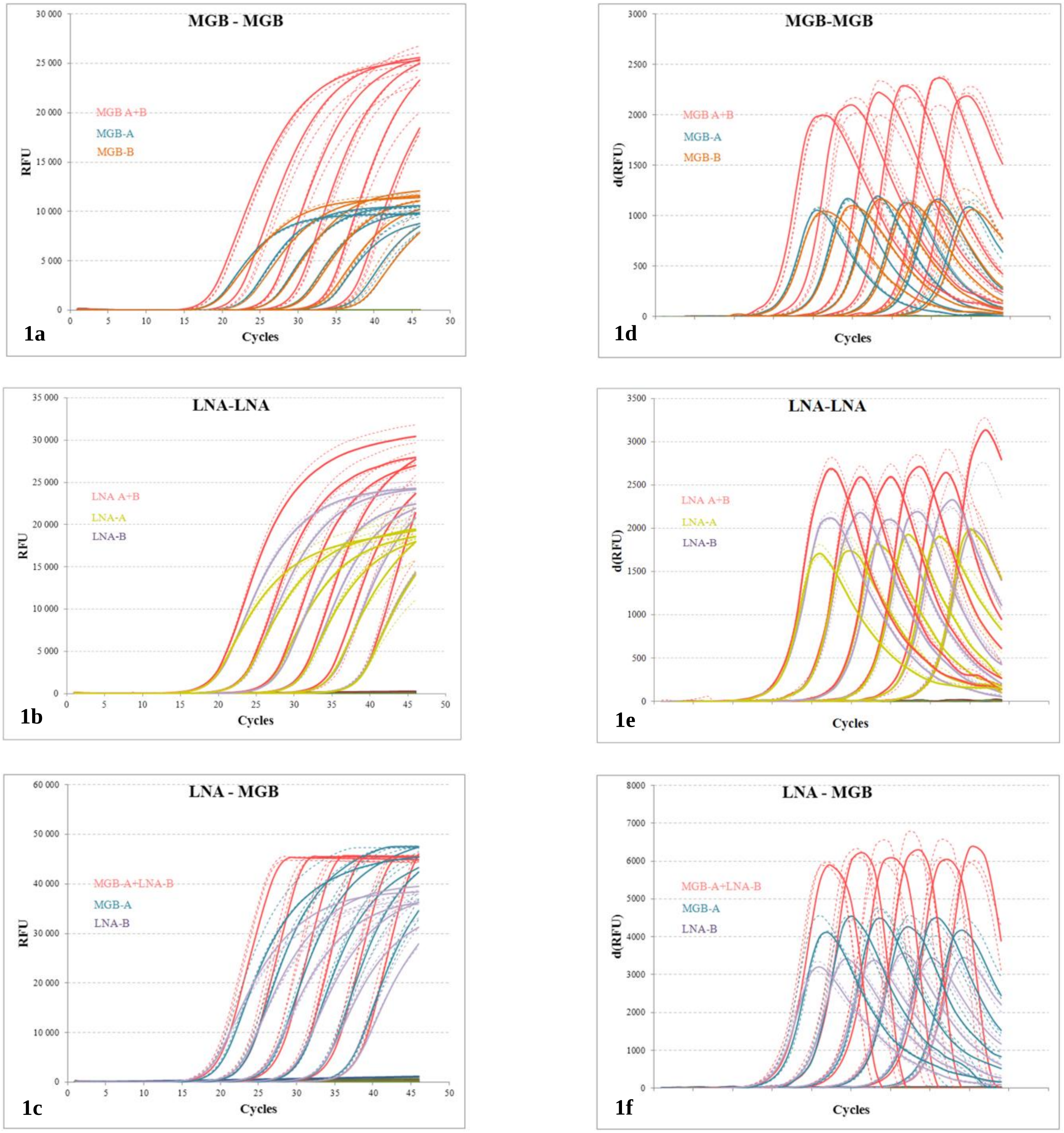

Figure S1.1. Dilution curve analysis of opposite dual-probe TaqMan qPCR assays.

Dilution curves **(1a-c),** arranged from 1e2 to 1e7 NA standard copies per µl of template in three replicates, of the MGB-MGB, LNA-LNA, and LNA-MGB dual-probe assays in opposite probe orientation and their first derivatives **(1d-f)**. The dual probe assays are highlighted in red and the corresponding single-probe counterparts in probe-specific colours. For clarity, certain curves were dashed. The probe sequences and their schematic representation and colouring are shown in Figure 1 and Table 1 in the article.

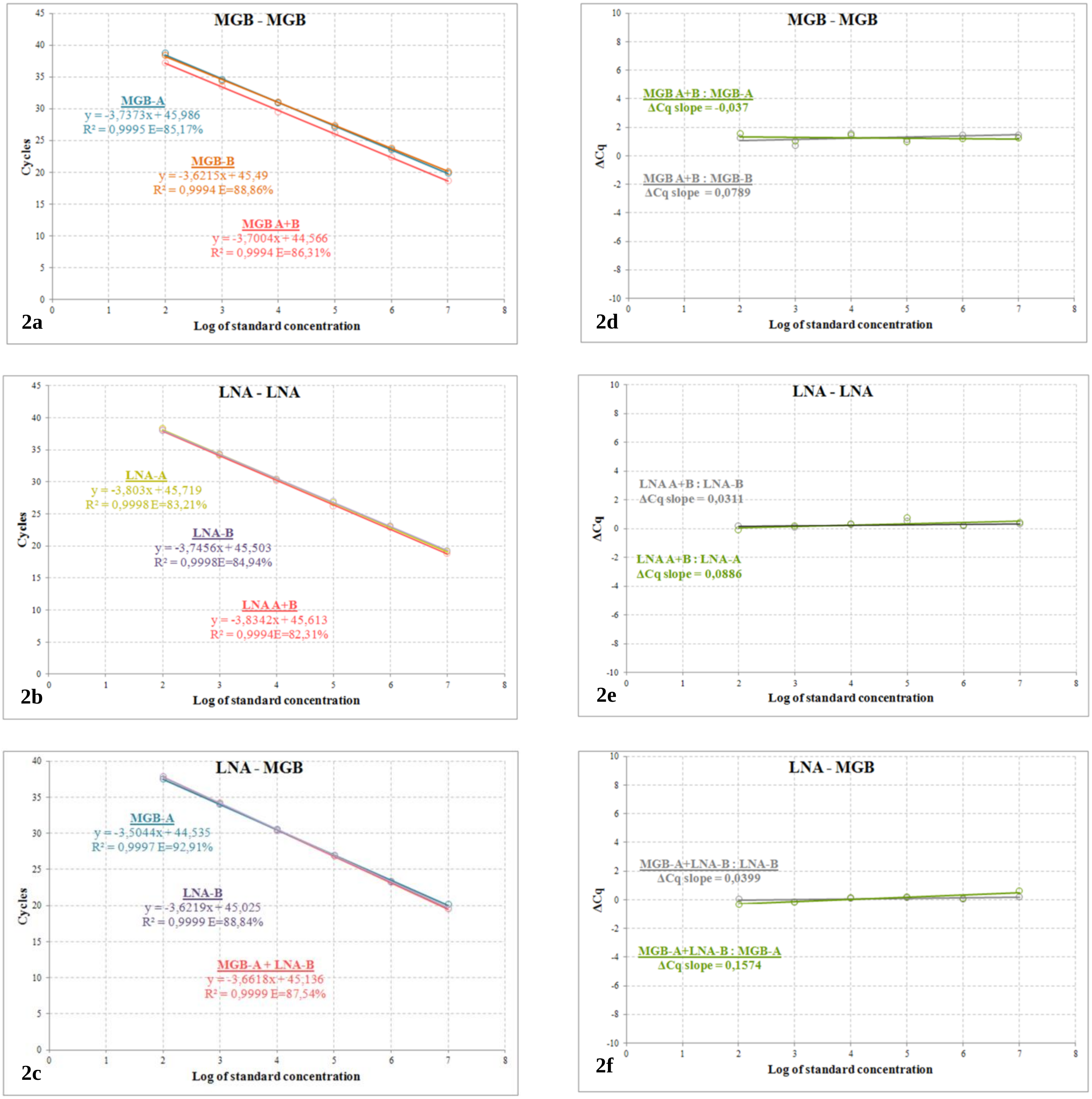

Figure S1.2. Calibration and ΔCq curve analysis of opposite dual-probe TaqMan qPCR assays.

Calibration **(2a-c)** curves of the MGB-MGB, LNA-LNA, and LNA-MGB dual-probe assays in opposite probe orientations. The curves were prepared for a dilution gradient from 1e2 to 1e7 NA standard copies per µl of template in triplicates. The dual-probe assays are highlighted in red and the corresponding single-probe counterparts in probe-specific colours. The probe sequences and their schematic representation and colouring are shown in Figure 1 and Table 1 in the article. The ΔCq curves **(2d-f),** representing the Cq differences between the dual-probe assay and the corresponding A and B assays at each concentration point, were coloured in green and grey, respectively.

Table S1.1: Relative fluorescence values of opposite dual-probe TaqMan assays. The table shows the differences in amplification curve fluorescence, expressed as percentage of the d(RFU) maxima and slopes, of the opposite dual-probe assays relative to the single-probe assays.

Probe combination	%d(RFU) and *%slope*											
	**1e2**		**1e3**		**1e4**		**1e5**		**1e6**		**1e7**	
	**A**	**B**	**A**	**B**	**A**	**B**	**A**	**B**	**A**	**B**	**A**	**B**
**Simple A+B**	48.8	30.2	47.4	32.5	48.8	32.8	49.4	30.0	47.4	30.0	50.3	31.8
	47.9	*31.6*	*45.7*	*31.2*	*46.9*	*30.2*	*47.7*	*30.5*	*46.8*	*29.6*	*50.4*	*33.4*
**MGB-A + Simple-B**	56.8	30.0	58.1	30.4	60.3	31.9	56.9	31.6	60.0	31.7	61.4	32.0
	56.2	*28.4*	*57.4*	*26.7*	*60.6*	*31.2*	*56.8*	*30.2*	*60.0*	*30.5*	*62.2*	*30.8*
**LNA-A + Simple-B**	33.4	39.8	40.3	43.2	40.7	46.2	36.8	45.8	39.1	45.4	39.9	46.5
	33.3	*41.7*	*39.1*	*42.2*	*41.3*	*49.7*	*36.4*	*48.4*	*40.0*	*46.5*	*38.6*	*47.8*
**MGB-A + MGB-B**	49.6	48.0	51.2	49.6	48.8	50.1	45.7	47.3	45.2	48.2	46.8	48.8
	53.0	*48.4*	*51.6*	*48.7*	*51.3*	*49.4*	*46.9*	*45.8*	*46.9*	*47.2*	*48.7*	*47.6*
**LNA-A + LNA-B**	37.3	26.5	31.5	17.6	30.2	20.6	24.9	27.5	31.1	17.7	35.9	21.3
	35.6	*25.6*	*31.1*	*15.2*	*33.9*	*20.3*	*20.3*	*17.2*	*28.6*	*14.9*	*34.3*	*19.9*
**MGB-A + LNA-B**	30.9	42.7	30.7	43.1	30.9	43.8	25.2	45.6	27.4	45.2	27.5	45.6
	31.6	*43.6*	*30.6*	*43.8*	*30.4*	*43.8*	*25.6*	*47.6*	*30.3*	*45.4*	*28.9*	*46.2*

Table S1.2: Repeatability of opposite dual-probe TaqMan assays. The table shows the intra-assay Cq variation of the tested probe combinations in opposite orientations.

Probe combination	Mean Cq±SD		
	**1e2**	**1e4**	**1e6**
**Simple A+B**	36.27±0.10	30.02±0.08	22.12±0.21
**MGB-A + Simple-B**	37.03±0.20	29.48±0.10	22.43±0.13
**LNA-A + Simple-B**	38.00±0.05	30.43±0.20	23.13±0.11
**MGB-A + MGB-B**	37.19±0.26	29.45±0.20	22.40±0.41
**LNA-A + LNA-B**	38.62±0.08	31.28±0.14	23.74±0.13
**MGB-A + LNA-B**	37.81±0.32	30.37±0.19	23.22±0.31

Table S1.3: Reproducibility of opposite dual-probe TaqMan assays. The table shows the inter-assay Cq variation of the tested probe combinations in opposite orientation.

Probe combination	Mean Cq±SD %RSD		
	**1e2**	**1e4**	**1e6**
**Simple A+B**	36.60±0.67 1.83%	30.13±0.37 1.24%	22.55±0.40 1.79
**MGB-A + Simple-B**	36.81±0.39 1,06%	29.87±0.40 1.34%	22.61±0.37 1.62%
**LNA-A + Simple-B**	37.60±0.62 1.64%	30.64±0.54 1.75%	22.99±0.52 2.25%
**MGB-A + MGB-B**	37.74±0.60 1,62%	29.36±0.24 0.80%	22.30±0.29 1.29%
**LNA-A + LNA-B**	38.64±0.28 0,74%	31.23±0.35 1,13%	23.48±0.33 1.39%
**MGB-A + LNA-B**	37.81±0.32 1.88%	30.37±0.19 0.84%	23.22±0.31 2.36%

Evaluation of TaqMan qPCR System Integrating Two Identically Labelled Hydrolysis Probes in Single Assay

Alexander Nagy[1,3*], Eliška Vitásková[1], Lenka Černíková[1], Vlastimil Křivda[1,2], Helena Jiřincová[3], Kamil Sedlák[2], Jitka Horníčková[2], Martina Havlíčková[3]

[1] State Veterinary Institute Prague, Laboratory of Molecular Methods, Prague, 16503, Czech Republic

[2] State Veterinary Institute Prague, Department of Virology and Serology, Prague, 16503, Czech Republic

[3] National Institute of Public Health, National Reference Laboratory for Influenza, Prague, Czech Republic

Supplementary Information 2. Results of Dual-probe TaqMan qPCR Assays with Tandem Probe Orientations.

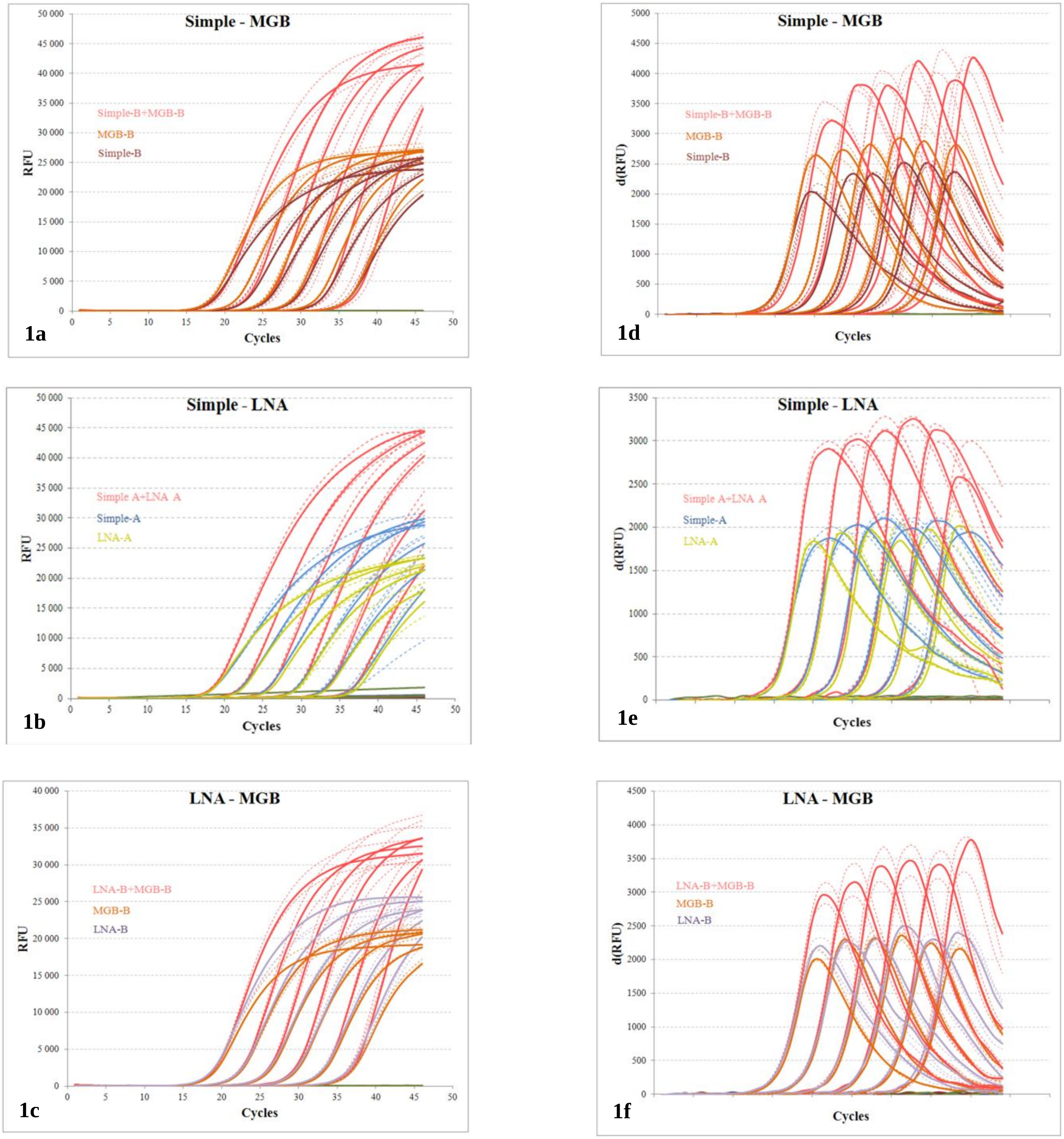

Figure S2.1. Dilution curve analysis of tandem dual-probe TaqMan qPCR assays.

Dilution curves **(1a-c),** arranged from 1e2 to 1e7 NA standard copies per µl of template in three replicates, of the simple-MGB, simple-LNA, and LNA-MGB dual-probe assays in tandem orientations and their first derivatives **(1d-f)**. The dual-probe assays are highlighted in red and the corresponding simple-probe counterparts in probe specific colours. For clarity, certain curves were dashed. The probe sequences and their schematic representation and colouring are shown in Figure 1 and Table 1 in the article.

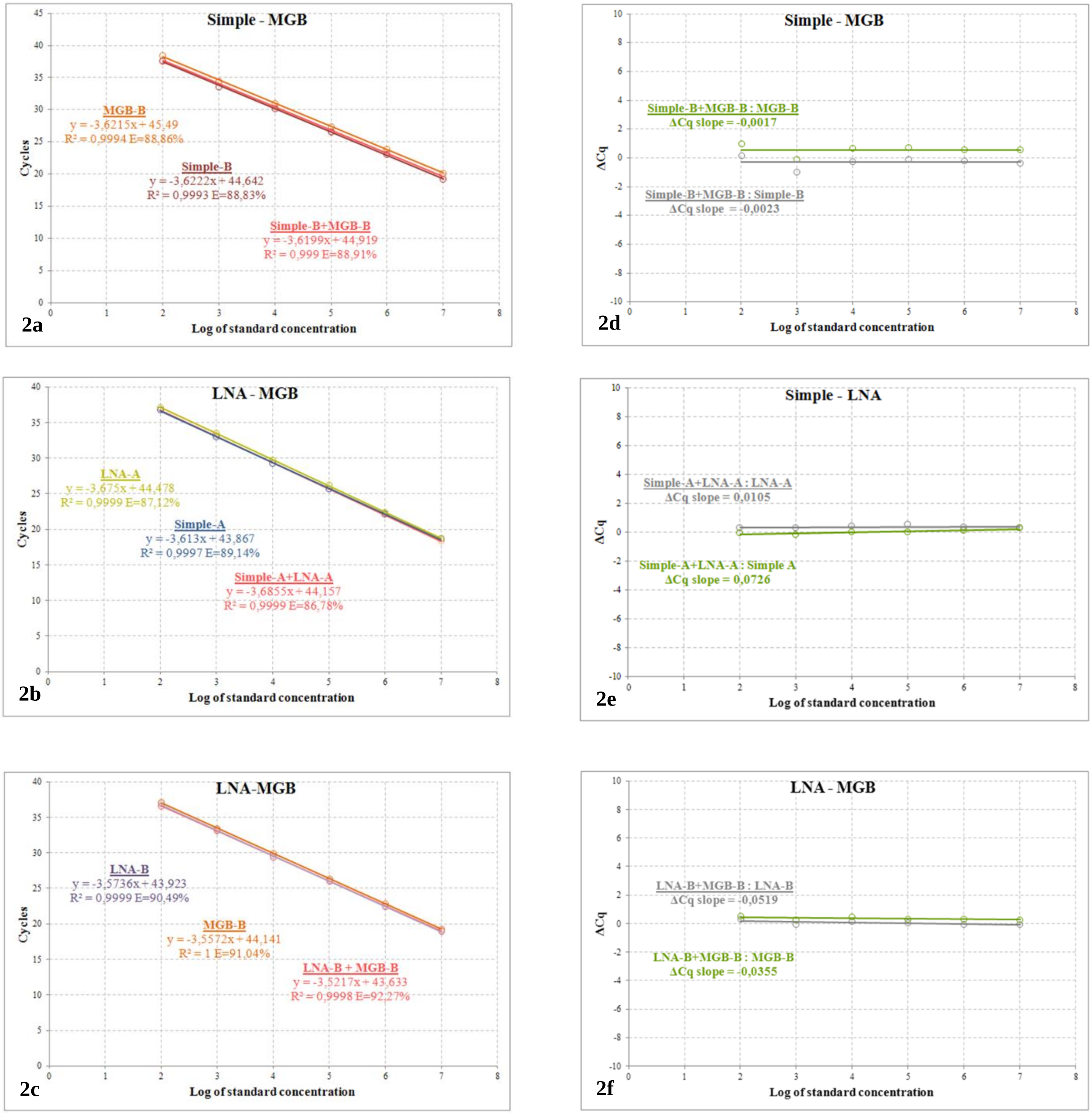

Figure S2.2. Calibration and ΔCq curve analysis of tandem dual probe TaqMan qPCR assays.

Calibration **(2a-c)** curves of the simple-MGB, simple-LNA, and LNA-MGB dual-probe assays in tandem orientations. The curves were prepared for a dilution gradient from 1e2 to 1e7 NA standard copies per µl of template in triplicates. The dual-probe assays are highlighted in red and the corresponding single-probe counterparts in probe specific colours. The probe sequences and their schematic representation and colouring are shown in Figure 1 and Table 1 in the article. The ΔCq curves **(2d-f),** representing the Cq differences between the dual probe assays and the corresponding single A and B assays at each concentration point, were coloured in green and grey, respectively.

Table S2.1: Relative fluorescence values of tandem dual-probe TaqMan assays. The table shows the differences in amplification curve fluorescence, expressed as percentage of the d(RFU) maxima and slopes, of the tandem dual-probe assays relative to the corresponding single-probe assays.

Probe combination	%d(RFU) and *%slope*											
	1e2		1e3		1e4		1e5		1e6		1e7	
	**A**	**B**	**A**	**B**	**A**	**B**	**A**	**B**	**A**	**B**	**A**	**B**
**Simple-B + MGB-B**	43.2<br>*43.4*	32.7<br>*32.8*	39.0<br>*37.8*	27.4<br>*27.3*	38.0<br>*37.9*	27.4<br>*29.2*	39.7<br>*40.5*	28.1<br>*27.6*	39.6<br>*41.2*	27.9<br>*28.8*	37.8<br>*37.8*	22.2<br>*20.0*
**Simple-A + LNA-A**	39.8<br>*39.7*	26.4<br>*29.0*	34.5<br>*32.8*	37.4<br>*38.5*	36.6<br>*36.2*	38.5<br>*40.5*	32.8<br>*32.7*	38.9<br>*42.0*	33.9<br>*32.7*	35.6<br>*39.0*	35.1<br>*34.6*	37.3<br>*38.0*
**LNA-B + MGB-B**	39.7<br>41.3	34.2<br>33.7	33.6<br>34.0	32.3<br>31.9	32.3<br>34.5	30.1<br>29.6	30.5<br>30.4	32.3<br>33.3	28.4<br>30.1	28.6<br>28.4	31.4<br>32.5	25.3<br>25.7

Table S2.2: Repeatability of tandem dual-probe TaqMan assays. The table shows the intra-assay Cq variation of the tested probe combinations in tandem orientations.

Probe combination	Mean Cq±SD		
	**1e2**	**1e4**	**1e6**
**Simple-B + MGB-B**	37.47±0.56	30.36±0,53	23.28±0.17
**Simple-A + LNA-A**	36.82±0.14	29.30±0,08	22.08±0.08
**LNA-B + MGB-B**	36.55±0.25	29.42±0,06	22.47±0.03

Table S2.3: Reproducibility of tandem dual probe TaqMan assays. The table shows the inter- assay Cq variation of the tested probe combinations in tandem orientations.

Probe combination	Mean Cq±SD<br>%RSD		
	**1e2**	**1e4**	**1e6**
**Simple-B + MGB-B**	37.01±0.52<br>1.40%	29.98±0.42<br>1.41%	22.85±0.36<br>1.60%
**Simple-A + LNA-A**	36.90±0.22<br>0.61%	29.57±0.23<br>0.79%	22.20±0.14<br>0,65%
**LNA-B + MGB-B**	37.10±0.53<br>1.42%	29.61±0.19<br>0.63%	22.23±0.18<br>0.81%

Evaluation of TaqMan qPCR System Integrating Two Identically Labelled Hydrolysis Probes in Single Assay

Alexander Nagy[1,3*], Eliška Vitásková[1], Lenka Černíková[1], Vlastimil Křivda[1,2], Helena Jiřincová[3], Kamil Sedlák[2], Jitka Horníčková[2], Martina Havlíčková[3]

[1] State Veterinary Institute Prague, Laboratory of Molecular Methods, Prague, 16503, Czech Republic

[2] State Veterinary Institute Prague, Department of Virology and Serology, Prague, 16503, Czech Republic

[3] National Institute of Public Health, National Reference Laboratory for Influenza, Prague, Czech Republic

Supplementary information 3. Evaluation of Dual-probe TaqMan qPCR Assays on Real Specimens.

Table S3.1: The primer and probe sequences used for the demonstration of dual-probe TaqMan qPCR utility in diagnostic microbiology.

Assay	Oligonucleotide	Sequence 5'→3'	Label	Modification	Orientation
**EAV**	Forward primer[32]	GTA CAC CGC AGT TGG TAA CA	---	---	opposite
	Reverse primer[32]	ACT TCA ACA TGA CGC CAC AC	---	---	
	Probe 1[32]	TGG TTC ACT CAC TGC AGA TGC CGG	FAM-BHQ1	simple	
	Probe 2[33]	TTG CGG ACC CGC ATC TGA CCA A	FAM-BHQ1	simple	
**CPV-2**	Forward primer[30]	TGG AAC TAG TGG CAC ACC AA	---	---	tandem
	Reverse primer[31]	CAA CCT CAG CTG GTC TCA TAA TAG T	---	---	
	Probe 1[30]	CAG GTG ATG AAT TTG CTA CAG G	FAM-BHQ1	simple	
	Probe 2[31]	ATG GGA AAT ACA AAC TAT AT	FAM-NFQ	MGB	

The colour scheme corresponds with the Figures and also in Figure 5 in the article.

CPV-2 (tandem simple-MGB)

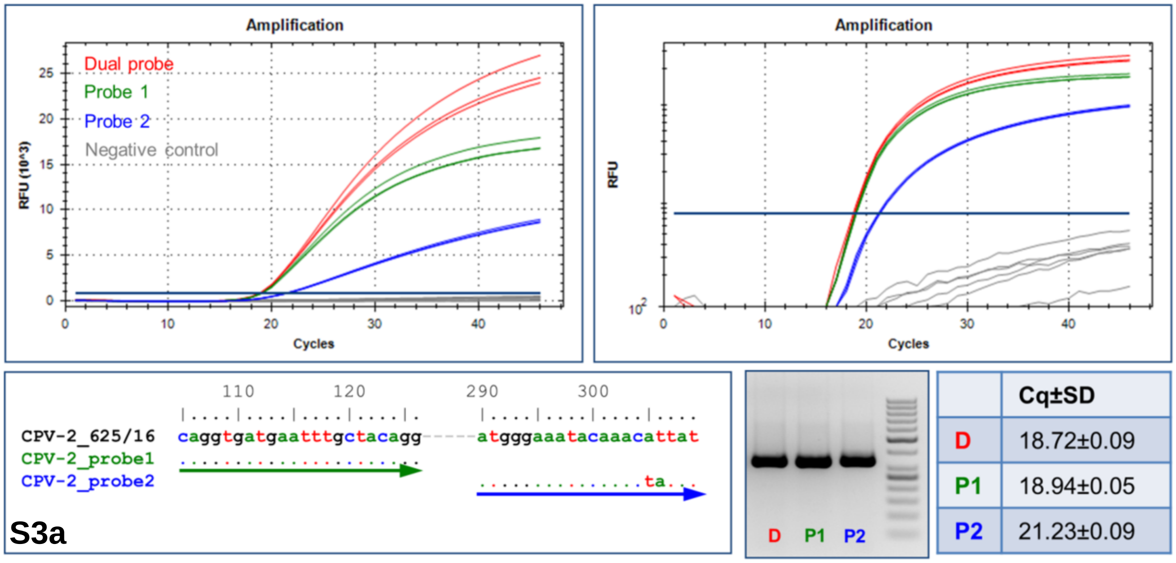

	Cq±SD
D	18.72±0.09
P1	18.94±0.05
P2	21.23±0.09

CPV-2 (tandem simple-MGB)

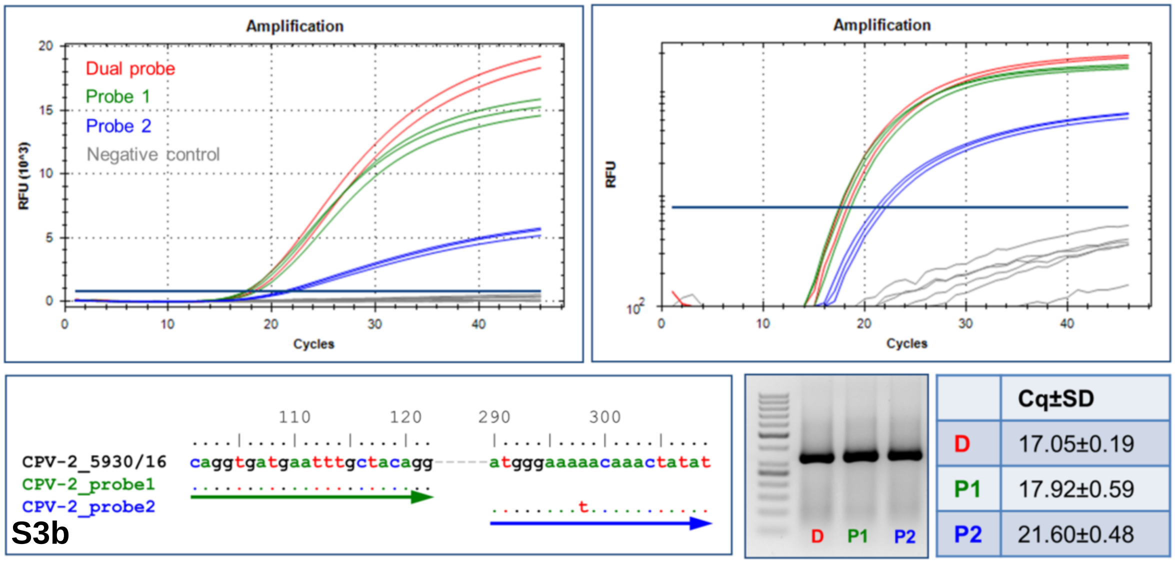

	Cq±SD
D	17.05±0.19
P1	17.92±0.59
P2	21.60±0.48

Figure S3. Demonstration of Utility of Dual-probe TaqMan qPCR in Diagnostic Microbiology

In these examples, the CPV-2_probe2 held two differences at the 3’ end (**a**) or a single change in the middle (**b**), relative to the CPV-2 template strain. The CPV-2_probe1 sequence is identical. As seen in both cases, the mutations influenced the probe binding, which was accompanied by a signal decline leading to flat curves, as well as increased Cq values. The common effect of both mutations led to probe failure, as shown in Figure 5a in the article.

EAV (opposite simple-simple)

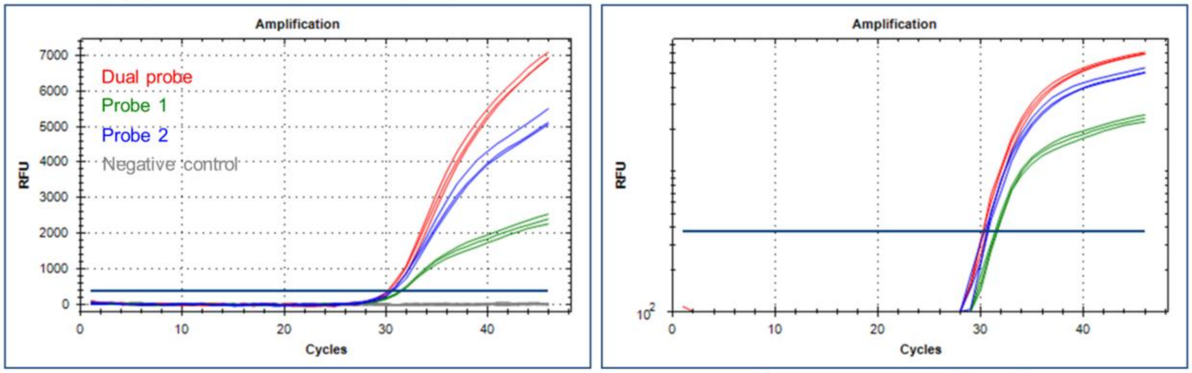

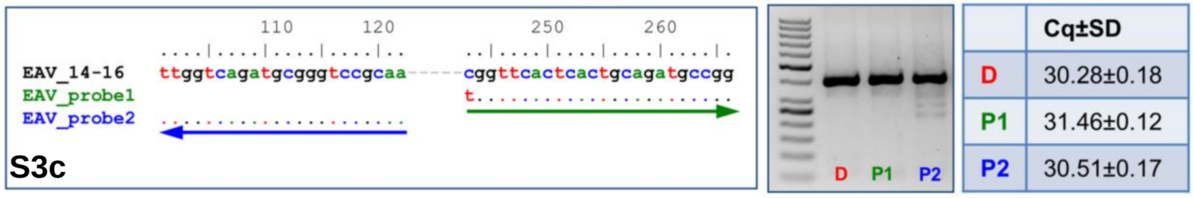

	Cq±SD
D	30.28±0.18
P1	31.46±0.12
P2	30.51±0.17

In the last example, the EAV_probe1 differed from its template in a single position located at the extreme 5’ end (**c**). The mutation weakened the probe binding, apparently due to interference with hydrolysis starting from the 5’ end. The probe was able to tolerate an additional change, as indicated in Figure 5b in the article.
